# American Veterinary College

**Published:** 1890-04

**Authors:** 


*American Veterinary College.*—The fifteenth annual commencement exercises of the American Veterinary College were held at Chickering Hall, New York, Wednesday, March 5th, 1890. Professor F. D. Weisse, M.D., president of the college faculty, presided, and among the members of the faculty who accompanied him on the stage were Professors J. A. Leighton, E. J. Brush, W. J. Coates, A. J. Dodin, E. J. Ryder, R. R. Bell, O. D. Pomeroy, C. A. Doremus, J. L. Robertson, A. W. Stein and A. Liautard, dean of the faculty.

The exercises were opened by the Rev. Otto Arnold, who offered prayer, after which the members of the graduating class received their diplomas from the President conferring the degree of Doctor of Veterinary Surgery. The following is the list of graduates: Charles Bruce Ainsworth, Greensburg, Ind.; Albert Fay Becker, Hemlock Lake, N. Y.; William Henry Berkmeyer, N. Y.; Henri Brister, N. Y.; Joseph Dodge Burtis, Jamaica, N. Y.; Edward John Creely, San Francisco. Cal.; Edward Francis Coyle, N. Y.; George Howard Davison, Ph. B., B.S.A., N. Y.; Mayhar Wigton Drake, N. Y.; Nathan Morroe Drake, N. Y.; George Eighmy, North Chatham, N. Y.; John Ernst, Jr., Valley Falls, Kan.; William Elsworth Groff, Massillon, Ohio; John Hall, West Union, N. J.; Christian D. Hartman, Manheim, Pa.; Henry Edward Holden, Springfield, Mass.; Edward Franklin Koehler, Easton, Pa.; Wesley Levi Labaw, Stoutsburg, N. J.; Frederick William Menhennitt, Champion, Mich.; Richard Middleton, A.B., Philadelphia, Pa.; John Robert Mitchell, Princeton, Ind.; Martin Arthur Morris, Lowell, Mass.; Archer Edward Parry, Denver, Col.; Frederick William Peniston, Bermuda, W. I.; Edwin Ross Ogden, Newark, N. J.; Thomas Henry Ripley, Newark, N. J.; Herbert Sheldon Sackett, Brooklyn, N. Y; Edward Lincoln Sander, N. Y.; Samuel Wesley Schuppan, Jersey City, N. J.; Elmer Kautz Shaub, Lancaster, Pa.; Eckley Rayner Storrs, Mansfield Centre, Conn.; Thomas Lawrence Swift, Falmouth, Mass.; Louis John Toussaint, Milwaukee, Wis.; George Vernon Towne, Thompson, Conn.; Seward Clarence Tremaine, Baddeck, Nova Scotia; Frederick William Turner, N. Y.; John Nicholas Wittpenn, N. J.

The class officers were: President, A. F. Becker; vice-president, A. D. Van Siclen; secretary, T. L. Swift; treasurer, J. Ernst, Jr.; class historians, C. B. Ainsworth and E. K. Shaub; marshal, W. B. Morris.

The following prizes were awarded by Professor C. A. Doremus;

Gold medal for the highest proficiency, to Richard Middleton, A. B., Philadelphia.

Gold medal presented by the faculty for the best practical examination passed before a committee of veterinary surgeons, to Edward John Creely, San Francisco, Cal.

A set of medical books for the second best examination passed before the same committee to Albert Fay Becker, Hemlock Lake, N. Y.

A case of dissecting instruments, presented by College Alumni Association, for the best written and defended paper read at the meetings of the Association during the term, to Joseph Dodge Burtis, of Jamaica, L. I.

A pocket case of instruments, presented by Professor Liautard for the best preparation in surgical anatomy, to Edward Francis Coyle, N. Y.

A communication was read from the French Minister of Agriculture by Monsieur Y. Dupas of the French Consulate, stating that the degree conferred by the college would be recognized by the French veterinary colleges.

The valedictory was delivered by W. E. Groff, of the graduating class. The Hon. J. R. Brady made an address pointing out the responsibilities and opportunities presented in the professional career of the graduates.

